# Atmospheric Pressure

**Published:** 1862-04

**Authors:** W. H. Atkinson


### ATMOSPHERIC PRESSURE.

BY DR. W. H. ATKINSON.

Atmospheric Pressure acts on all exposed sides of bodies with equal or nearly equal force, hence in the quiescent state of these it is not appreciable; and even in certain directions of application of force, it is not appreciable, these lines are at right angles to the line of gravitation. But the moment a movement takes place in an upward direction or in a downward direction in a line with the force of gravity, it is at once apparent that the atmosphere does exert its well known power of some 14 ℔ to the square inch of surface acted upon.

The question of suction and atmospheric pressure, being identical or diverse, is like many other questions in philosophy dependent upon just what is meant by the term used; to many loose and careless speakers, they are identical; while to the fractional and crotchety hair splitter, they are "no whit alike." So take one or other of these definitions we shall not advance so long as we array one against the other.

Mutual dependence of fact and philosophy, ponderable and imponderable, secular and sacred, must be recognized to enable us to rise above not merely branches and separations, but actual controversies waged at every point of contact along the whole line of ideal and practical labors. Be assured that difference in apprehension is the only difference among scientific and truthful minds.

Terms are useful when tersely and distinctly conveying the intent of speaker or writer; but when used to darken council, and set up for one against another specific definition, they are not helps to understanding of a true philosophy. What divides the advocates of "*suction*" on the one hand and "*atmospheric pressure*" on the other, is fractional apprehension of each others meaning, for both do *mean* practically just the same thing.

Let us see how they differ; and then lay aside selfish adhesion to any pet phrase and come into the blessed light that shines forth calling to us simple ones to "be wise."

Atmospheric pressure becomes suction at the point of action.

We may prove this in the many hackneyed methods and stale examples, of boards or other slab-like bodies, not ignoring the inevitable "scientific toy," the boys wet leather with a string in the center of the disk.

It has already been stated that bodies moved horizontally gave no evidence of the presence of this force. Neither does it display itself in the case of the leather disk if it be moved transversely to the line of gravitation, or by lifting at its edge. But apply the force of lifting at the centre of disk so as to approximate the production of a vacuum and then it is clearly thus brought into action, and very apparent. It is not necessary to draw the brick through the disk to demonstrate the act of suction more than it is necessary to swallow your mint julep to demonstrate that it traversed the tube to the oral cavity by a true suction.

He who can not see that it is the same principle which acts in the one case as the other is to be patiently dealt with and digged about and watered with the patient love of the hand-maid of truth, demonstrable fact, until conviction once finds lodgment in his perceptive ability, when he will at once be converted from his fractional and stubborn selfish ways and joyously join to swell the anthem to a wholeness of knowledge, no more to go back to the chrysalis state of mind.

Shut out the atmosphere by any means from coming between any two surfaces impervious thereto and exhaust the intermediate space or approximation to space, and, you have control of this power for mechanical purposes, whether in or out of the mouth. Many a very imperfect fit may be rendered practically useful by filling the cup like upper surface with fluid before putting it into the mouth, thus avoiding the presence of air between plate and gum by the denser medium which if not all drawn out, will allow adhesion by the support of the column of free air in the oral chamber (mouth). until by repeated efforts at sucking out the fluid, the soft parts will have so adapted themselves as to retain the piece in "situ" firmly in these cases only approximating a fit.

The question of serviceability of cavities or, as they are generally called, "air chambers" may be set down in the category of advocates and opposers as occupying the same general stasis of suction and atmospheric pressure, only a stumbling block and rock of offence to those who tary in the fractional expression of the philosophy involved. The advocates exaggerating the size and depth of the cavity beyond any necessity for attaining the best results, while the opponents actually do approximate the production of a cavity by nicely easing off the plate over the central portion of the palatine surface where the integument is so likely to be dense and resistant, thus securing the approximation to the production of a vacuum, the very point at which atmospheric pressure or suction is surely and easily attainable even without the "perfection of adaptation" so much insisted on as necessary to the proper retention of the piece in place.

So we may be assured that a sort of a middle ground between other advocates of extreme measures of "perfect fits" on the one hand, and the reckless carelessness and hardihood of guessing at the form of the alveolar borders, making a piece by throwing it hastily and roughly together and inserting it with the assurance to the patient that the mouth will adapt itself to it.

